# Protein production in *Escherichia coli* is guided by the trade-off between intracellular substrate availability and energy cost

**DOI:** 10.1186/s12934-019-1057-5

**Published:** 2019-01-17

**Authors:** Alexander Nieß, Martin Siemann-Herzberg, Ralf Takors

**Affiliations:** Institute of Biochemical Engineering, University of Stuttgart, Stuttgart, Germany

**Keywords:** Translation elongation, Heterologous gene expression, Codon-adaptation, Cell-free protein synthesis, Translation optimization

## Abstract

**Background:**

In vivo protein formation is a crucial part of cellular life. The process needs to adapt to growth conditions and is exploited for the production of technical and pharmaceutical proteins in microbes such as *Escherichia coli*. Accordingly, the elucidation of basic regulatory mechanisms controlling the in vivo translation machinery is of primary interest, not only to improve heterologous protein production but also to elucidate fundamental regulation regimens of cellular growth.

**Results:**

The current modeling analysis elucidates the impact of diffusion for the stochastic supply of crucial substrates such as the elongation factor EFTu, and tRNA species, all regarded as key elements for ensuring optimum transcriptional elongation. Together with the consideration of cellular ribosome numbers, their impact on the proper functioning of the translation machinery was investigated under different in vivo and in vitro conditions and utilizing the formation of non-native GFP and native EFTu as target proteins. The results show that translational elongation was diffusion limited. However, this effect was much more pronounced for the translation of non-native proteins than for the formation of codon-optimized native proteins.

**Conclusions:**

Cellular ATP requirements constrain the options of improving protein production. In the case of non-native protein sequences, an optimized tRNA supply may be the most economical solution, as cells necessarily have to invest in ATP-costly ribosome synthesis to boost translation and increase growth rates.

## Background

The translation of mRNA-encoded information in executing proteins is a crucial part of cellular life and takes place at the ribosomes. Studies investigating the in vivo limits of ribosomal translation are of primary interest. They offer insights into fundamental questions such as whether or not translation rates limit maximum cellular growth. Additionally, they may give answers to technical problems that often occur when recombinant proteins are produced in microbial hosts.

*Escherichia coli* is well known as a prominent producer of technical and pharmaceutical proteins, and contributes a large share of the 30% biopharmaceuticals that are produced by microbial hosts [20]. This bacterium shows characteristic drawbacks such as stalling translation. The phenomenon hampers heterologous protein formation [15] and is usually attributed to rarely used codons [21, 24]. Optimizing the gene sequence is an often applied empirical countermeasure [17]. However, model-based approaches considering mRNA secondary structure or the codon adaptation index (CAI, [21]) still need refinement [23]. Conversely, *E. coli* has the highest growth rate of the industrially used heterologous protein producers (0.7 to 1.7 per hour, depending on the growth medium), which hints to a large translational capacity. Assuming that high growth rates also indicate high translation capacities, ultra-fast growers such as *Vibrio natriegens* (maximum growth rate: 4.4 per hour [9]; may even offer higher translational rates compared to *E. coli*.

This study was performed to characterize the in vivo translation capacity of *E. coli*, with particular focus on the availability of translation substrates such as the elongation factor EFTu, or tRNAs, or resulting ternary complexes of EFTu and tRNAs. Because the substrates have low intracellular concentration, we implemented stochastic modeling to mirror the putative impact of the rare supply events in the cells.

**Fig. 1:**
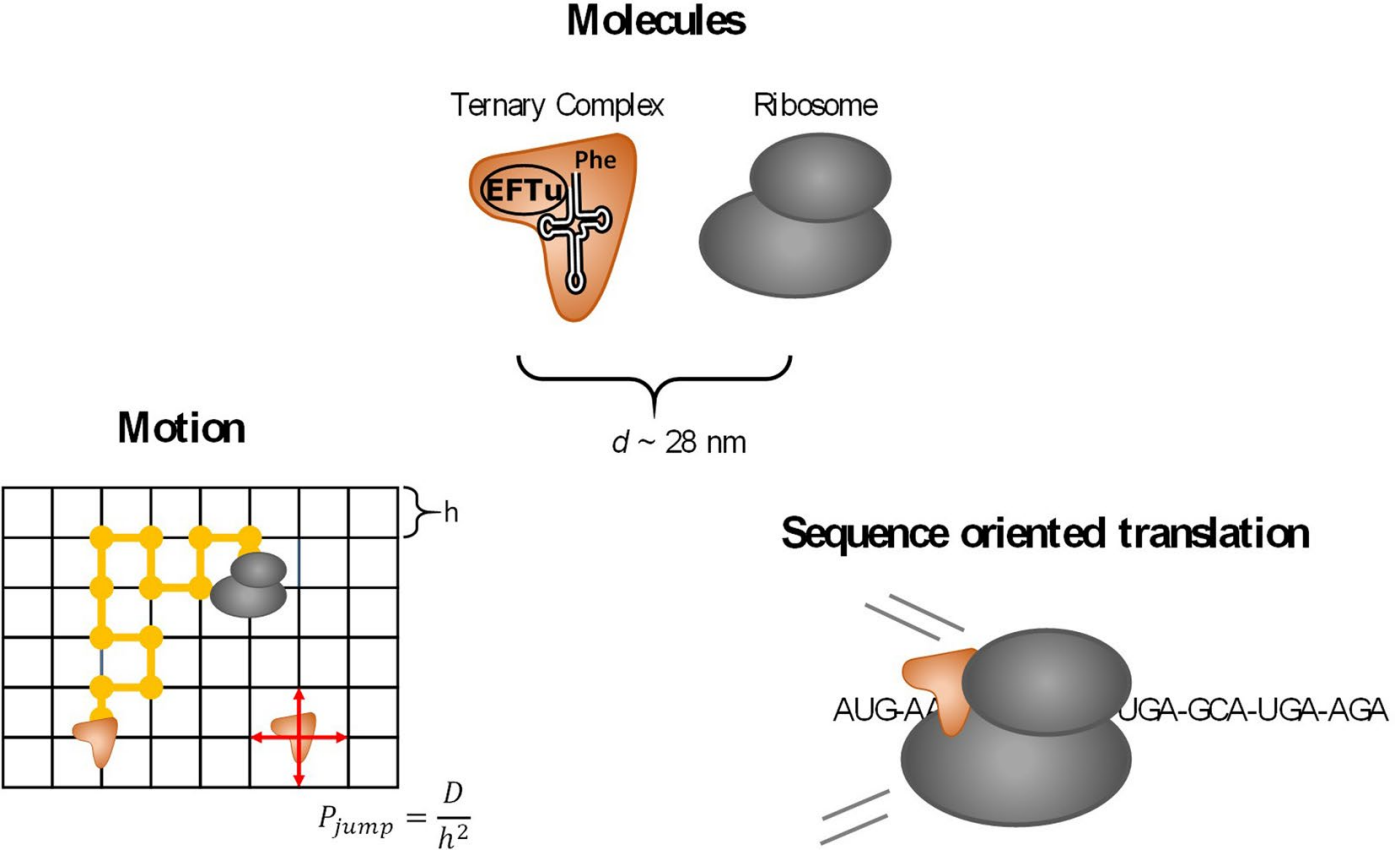
Overview of the key elements regarded in this study. (1) Ternary complexes and ribosomes as reaction partners, (2) sequence oriented translation elongation and (3) diffusion driven motion of molecules

In *E. coli*, 46 different tRNA species have been identified so far [5]. Assuming that (1) approximately 13,500 ribosomes are required per cell at a growth rate of 0.7 per hour and (2) 80% of ribosomes are actively translating, and (3) the elongation rate is 16 amino acids per ribosome per second, then more than 170,000 amino acids per second are needed for protein synthesis [2]. This number equates to an individual tRNA turnover time of about 0.67 s. Noteworthy is that the cycling time comprises crucial steps such as the release of tRNA from ribosomes, transport for recharging at aminoacyl tRNA synthetases, and back transport to ribosomes. Since convective flow is absent within cells, tRNA transport should be driven by diffusion. Consequently, a comprehensive model linking stochastic transport with mechanistic translation modeling [14] was created for investigating the sensitivity of tRNA supply on translation capacities and, as a further step, to deduce detailed understanding of cellular strategies to minimize energy expenses for translation. Figure 1 gives a schematic overview of these model blocks.

## Results

### Factors affecting in vitro and in vivo elongation times

Assuming that translation elongation is limited by the transport of ternary complexes towards the actively translating ribosomes, we established a model that describes diffusive motion of ternary complexes in a three-dimensional space. Furthermore, collisions of ternary complexes with tRNA-free ribosomes were defined as successful encounters resulting in instantaneous reactions. To minimize computational efforts, the reaction volume was set to 0.064 µm^3^ for preliminary studies, and time steps *t*_*enc,s*_ between two successful encounters were evaluated.

**Fig. 2:**
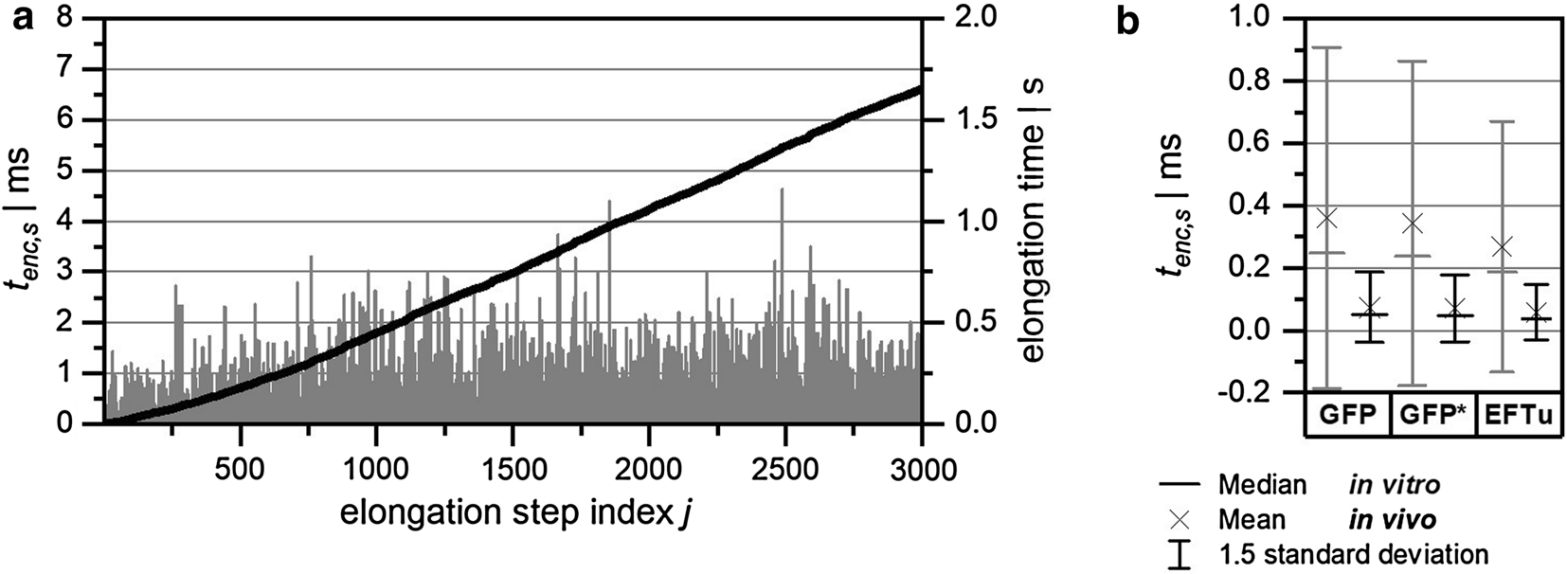
**a** Distribution of elongation step duration (*t*_*enc*,*s*_, bars) in the reaction volume and the resulting overall elongation time (sum of elongation step duration, black line) for the first 3000 elongation steps of GFP as the target sequence (in vivo conditions). **b** Evaluation of elongation step duration in vivo and in vitro for GFP, codon optimized GFP (GFP*), and EFTu as the target sequence

Figure 2a shows the distribution of *t*_*enc,s*_ for the first 3000 elongation steps with GFP as the target gene sequence in an in vivo scenario (concentrations according to a growth rate of 1.1 per hour). Additionally, the overall elongation time, calculated as the sum of *t*_*enc,s*_, is depicted. The course of the overall elongation time shows an increasing slope for the first 1000 elongation steps and a constant slope afterwards. The increasing slope reflects the not yet achieved steady-state of the translation machinery. Accordingly, the first 1000 elongation steps were omitted to remove the impact of the initial, random molecule distribution from the calculation of mean elongation times. Therefore, only elongation rates at stable steady-state conditions were evaluated.

In vitro and in vivo conditions differ severely because the first are typically diluted by a factor of 20 [14]. Therefore, the initial concentrations for the in vitro scenario were calculated as 1/20th (relative concentration = 0.05) of the in vivo scenario (relative concentration = 1). Impacts of reactant concentrations can be investigated comparing in vitro with in vivo conditions. Additionally, the effect of varying target gene sequences on elongation times was studied (Fig. 2b). In comparing in vivo with in vitro encounter times, in vitro times showed higher mean and median values, together with significantly broader standard deviations for all three investigated target sequences. Consequently, lower elongation rates were calculated for the in vitro scenario compared to those for in vivo conditions. On average, every 50–60 µs one translation elongation step took place for in vivo settings whereas 270–360 µs were found with in vitro concentrations, both using 0.064-µm^3^ reaction volume. Scaling the reaction volume to cellular conditions (1 µm^3^) reduced the in vivo time between two subsequent elongation steps to approximately 3–4 µs. Furthermore, the respective standard deviation was found to be much smaller under in vivo conditions.

The impact of the gene sequence can be assessed by comparing the common GFP sequence with a codon-optimized variant [14]. Here, the optimized variant shows a 6% higher overall translation rate. Applying the z-score analysis according to Paternoster et al. [16] and Cohen et al. [4] gives a z-score of 5.3 and a corresponding p-value < 0.001 with df = 3996 outlining the statistically sound difference between the slopes. However, comparing GFP with a native gene sequence such as the EFTu sequence, 35% increased elongation rates were identified for EFTu.

### Impact of dilution on elongation rates

Diffusive transport is highly dependent on the concentration of molecules. Therefore, we investigated the translation rate as a function of the concentration of the translation machinery. EFTu, tRNA, and ribosome levels were taken from the published in vivo values [2]. The relative concentrations of the three species were normalized with respect to the conditions at a 1.1 h^−1^ growth rate.

**Fig. 3:**
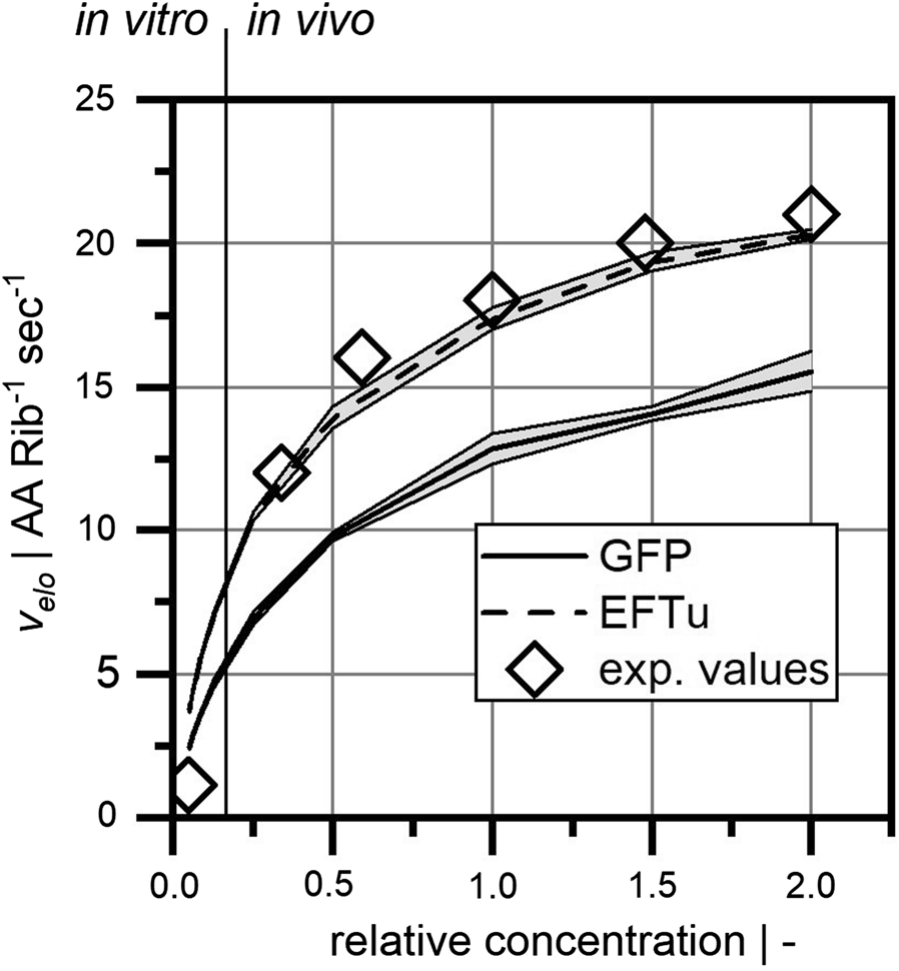
Ribosome specific elongation rate for diffusion-limited translation as a function of the relative concentration. The lines indicate simulation results with grey areas showing the standard error at each point). Here, the sequences for the target proteins EFTu and GFP were investigated. Experimental values (exp. values) were derived from Bremer and Dennis [2] for in vivo (growth rate > 0.2 per hour) and from Nieß et al. [14] for in vitro (growth rate < 0.2 per hour). Relative concentrations were calculated based on a growth rate of 1.1 per hour

Figure 3 shows the resulting average elongation rate in the reaction system for a broad range of concentrations and two different target proteins. EFTu represented a native gene sequence and GFP a non-native target sequence (non-optimized variant). Both sequences revealed increasing elongation rates with increasing normalized concentrations. Interestingly, elongation rates of GFP were always found to be significantly lower than elongation rates of the native EFTu sequence.

Under reference conditions (normalized concentration of 1.0, corresponding to a growth rate of 1.1 per hour), the model predicted elongation rates of 17.3 amino acids per ribosome and per second for the EFTu sequence compared to 18 amino acids per ribosome and per second [2] (calculated as quotient of total translation rate of all proteins divided by the number of actively translating ribosomes). Conversely, GFP only achieved approximately 12.5 amino acids per ribosome and per second, which is 27.8% less than that of the EFTu sequence. Diluting EFTu, tRNA, and ribosome levels to in vitro levels reduced elongation rates disproportionately to 21.5 and 19.1% of the elongation rate under reference conditions, respectively. To be precise, diluting reactants by a factor of 20 only caused elongation reduction by a factor of approximately 5. Elongation differences between native and non-native sequences narrowed to an equal extent.

### Control analysis

The impact of different concentration levels of ribosomes and ternary complexes on the resulting translation rate can be assessed by translational control analysis [25]. Here, the elasticity $\epsilon_{EFTu \cdot tRNA}$ is introduced, describing the sensitivity of the translation rate with respect to changing concentrations of ternary complexes (“substrate”). Similarly, the flux control coefficient (*FCC*) describes the impact of altered ribosome (“enzyme”) levels on the resulting translation rate.

$$\epsilon_{T3} = \frac{{\partial v_{TL} }}{{\partial c_{T3} }}\frac{{c_{T3} }}{{v_{TL} }}$$

$$FCC = \frac{{\partial v_{TL} }}{{\partial c_{R} }}\frac{{c_{R} }}{{v_{TL} }}$$

To investigate which components may increase the elongation rate, we evaluated elasticities and flux control coefficients for different concentrations. The concentration range was chosen on the basis of control scenarios under in vitro and in vivo conditions. Furthermore, we investigated GFP and EFTu as target sequences to identify the impact of codon optimization.

**Fig. 4:**
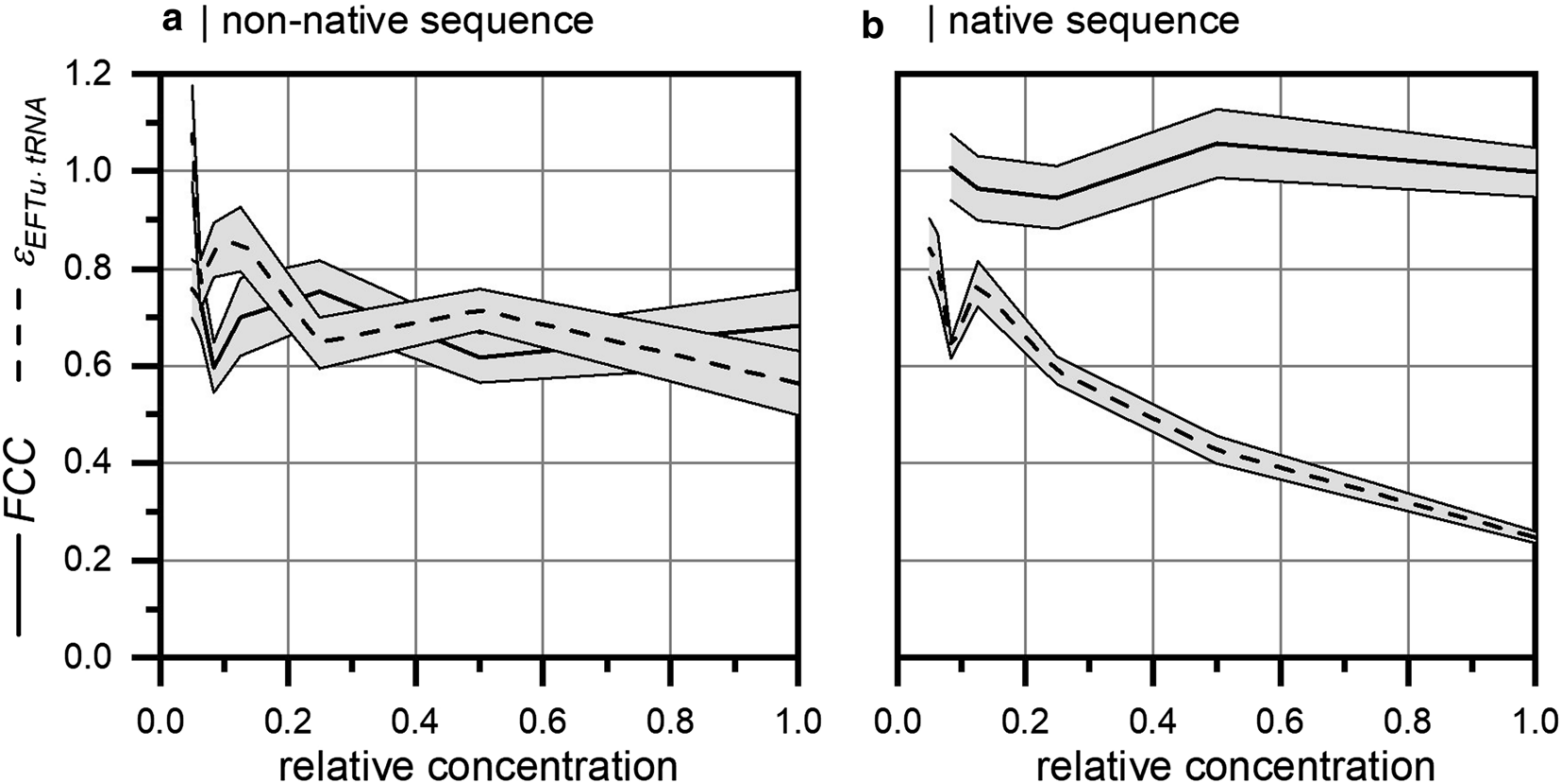
Control analysis of translation elongation for varying relative concentrations of the reaction system. Here, a non-native (GFP) and a native (EFTu) gene as target protein sequences were investigated. Elasticities $\epsilon_{EFTu \cdot tRNA}$ and *FCC* represent the sensitivity of the translation rate with respect to varying total ternary complex (substrate) and total ribosome (enzyme) concentration. Grey areas indicate the standard error at each point

Figure 4a shows the FCC and the elasticity of GFP as the target sequence. Regarding GFP as a target protein, *FCC* and $\epsilon_{EFTu \cdot tRNA}$ revealed constant values of approximately 0.7 for the whole concentration range. Accordingly, impacts of ternary complexes and ribosomes on the translation rate remained constant irrespective of their concentration. The translation of EFTu as the target sequence is shown in Fig. 4b. Here, *FCC* has a constant value of approximately 1.0, whereas the elasticity decreases with increasing concentration of the reaction system. This indicates that the translation rate scales linearly with the amount of ribosomes. However, the impact of the ternary complexes decreases with increasing relative concentrations.

Summarizing, elongations of non-native (GFP) and native (EFTu) protein sequences reveal strong dependencies on ribosomal availability irrespective of the concentration level. However, the sensitivity is much more pronounced for native sequences. Increasing ribosomal availability may be a general option for improving translation capacities. However, from a cellular perspective, this option comes with ATP expense for ribosome formation, which will be discussed in the next section.

### Improving translation with minimum ATP needs

Cellular options to improve translation rates may comprise increasing availabilities of the elongation factor EFTu, tRNA, and/or ribosomes. Costs for de novo synthesis of these molecules highly differ, which was important in analyzing ATP balancing. We evaluated cellular energy management by qualifying different scenarios of ATP spending for the synthesis of the three translation factors. Accordingly, 10, 50, and 100 million ATP molecules per reaction volume were investigated, which equaled 1, 5, and 10 fmol ATP per cell, respectively. Assuming ATP pool sizes in *E. coli* of 3.56 amol per cell [3] and an ATP turnover of 311 per minute [10], these ATP concentrations relate to the ATP production of 0.9, 4.5, and 9 min, respectively. Dividing these values by the doubling time of 38 min (at a growth rate of 1.1 per hour) reveals that the ATP concentrations correspond to 2.4, 12 and 24% of the overall ATP synthesis capacity.

**Fig. 5:**
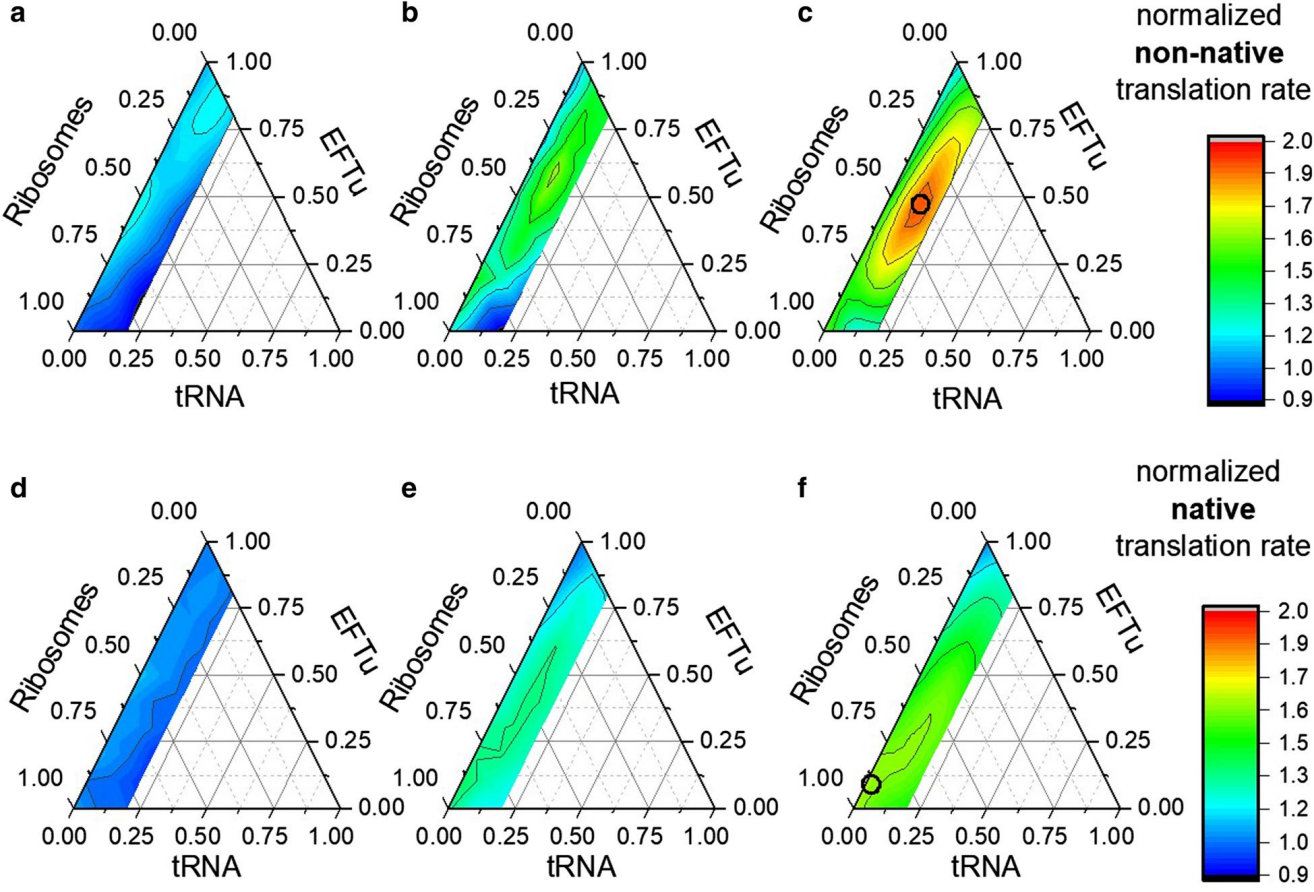
Distribution of ATP in tRNA, EFTu, and ribosomes, and the resulting translation rate (normalized to unaltered conditions) for the non-native (GFP) (**a**–**c**) and the native (EFTu) (**d**–**f**) protein target. The point of maximum translation rate was highlighted with black circles in panels **c** and **f**. The amount of ATP distributed was 1 × 10^7^ (**a**, **d**), 5 × 10^7^ (**b**, **e**) and 1 × 10^8^ (**c**, **f**) ATP molecules per reaction volume (approximately 1 M, 5 M, and 10 fmol ATP equivalents per cell, respectively)

Figure 5 comprises a set of six ternary diagrams depicting translation rates as a function of the native (EFTu) and non-native (GFP) target gene sequences and three ATP spending scenarios.

Greater ATP expenditures allowed higher translation rates. A 1.9-fold increased translation rate was achieved for the non-native gene sequence at 10 M ATP concentration, with ATP shares of 37.5% to ribosomes, 12.5% to tRNA, and 50% to EFTu, respectively. With the same amount of ATP, translation rates of the native target protein could be improved by 1.6-fold, with 90% of the ATP to ribosomes and 10% to the elongation factor EFTu.

## Discussion

### Factors affecting in vitro and in vivo elongation times

Microorganisms that show a fast growth rate inherently need a high capacity for protein biosynthesis to supply the increasing biomass with proteins. With increasing growth rates, *E. coli*, for example, reallocates existing translational capability towards the synthesis of proteins necessary for translation. During growth on minimal media, translation-associated proteins account for approximately 16 mass% of all proteins, whereas in complex media, the value increases up to 34% [12]. For comparison, transcription-associated proteins increase from 2.5 to 2.8 mass% with increasing growth rate. Under these rapid growing conditions, one of every two proteins expressed is related to translation. These values show that a fast growth of microorganisms is accompanied by the need for high translational capability.

Using a stochastic diffusion collision model to describe translational elongation allowed the calculation of elongation rates as a function of diffusive transport. We investigated a broad range of normalized concentrations and showed that this model was able to simulate published elongation rates within a very small error margin.

From this, we conclude that stochastic diffusion events play a key role in the total performance of the translation machinery. Translation rates of both native and non-native gene sequences heavily depend on the availability of translation substrates. Simulations shown in Fig. 3 reveal that translation improvements beyond the in vivo condition are even possible, provided that concentrations of the translation factors are substantially increased. Additionally, the proper composition of the primary mRNA sequence is of utmost importance. EFTu, as an example of a highly optimized native gene sequence, achieved approximately 30% higher elongation rates than non-native GFP. Apparently, the interaction of codon composition and tRNA distribution directly influences the elongation rate. In essence, codon optimization reflects harmonized needs of properly charged tRNAs. Short-term shortage of tRNAs is prevented, and unwanted translation stalling is avoided. In other words, when the limiting impact of rare tRNA diffusion is eliminated, short-term shortage of tRNAs is prevented, and unwanted translation stalling is avoided.

### Control analysis

EFTu forms ternary complexes with tRNAs, which represent major substrates of ribosomes. Accordingly, we investigated the sensitivity of the translation rate with respect to the availability of ternary complexes and ribosomes. Irrespective of the gene sequence, native or non-native, translation rates had a constant dependency on ribosome concentration, albeit at different levels. The increase of ribosome concentration is a general approach to increase translation rates, in particular when translation rates of native proteins needs to be improved. Strikingly, the translation of non-native gene sequences was amplified by increasing the availability of ternary complexes. We hypothesize that the non-optimized codon sequence caused shortages of distinct tRNA species, which then required compensation by concentration increase.

### Improving translation with minimum ATP needs

Increasing ribosome numbers provides a general strategy to increase translation rates of native and non-native sequences under in vivo conditions. However, cellular ATP expense to synthesize ribosomes is extremely high (108,461 ATP equivalents), whereas formation costs of tRNA and EFTu are lower by orders of magnitude (700 and 3565 ATP equivalents, respectively). Assuming that cellular decision-making is constrained by ATP needs, the cellular strategies to increase translation rates heavily depend on the gene sequence of the target protein. To be precise, translation of non-native proteins sequences can be improved by de novo synthesis of ATP-inexpensive ternary complexes. After a sufficient supply has been provided, the remaining ATP can be utilized for the de novo synthesis of ribosomes. In contrast, native target proteins already possess equilibrated tRNA needs, which requires cellular investments in expensive de novo ribosome synthesis.

Because native protein sequences are usually codon optimized by evolution, cells need to increase ribosome numbers for proportionally increasing translation rates. Therefore, increased growth requires equally increased ribosome numbers per cell. This statement is in agreement with early findings of Bremer and Dennis [2]. Cellular ribosome numbers increased disproportionately compared to tRNA and EFTu in *E. coli* with higher growth rates. Modeling results of this study fully support the experimental observations. The impact of ternary complex supply was much less pronounced under in vivo conditions than under in vitro conditions.

## Conclusions

The good accordance between simulated and experimental elongation rates indicates that diffusion of ternary complexes apparently is one of the key rate-limiting mechanisms during in vivo and in vitro translation. In particular, the production of heterologous proteins may benefit from this action by preventing shortages of rare tRNAs. Importantly, this does not only suggest that codon optimization is needed to achieve a balanced distribution of different tRNAs encoding the same amino acid; it also prevents repetitive codon sequences (e.g. for His-Tags) from causing intermediary dynamic shortages. By analogy, in vitro protein synthesis (cell-free protein synthesis) may benefit from adjusting concentrations of tRNAs properly in the bioreactors. Through evolution, gene sequences of native proteins are codon-optimized, thereby minimizing potential tRNA shortages. As a consequence, *E. coli* needs to invest in ATP-costly ribosome synthesis to improve translation and to accelerate growth. This underlines the common observation that increased growth rate is always linked to a proportional increase of cellular ribosome numbers.

## Methods

An overview of the parameters and variables are given in Table 1.

**Table 1:** Overview of symbols used in this study and their corresponding units

Symbol	Unit	Explanation
$\epsilon_{T3}$	Dimensionless	Elasticity of translation over the amount of ternary complexes
*FCC*	Dimensionless	Flux control coefficient
*v*_*TL*_	µM amino acids s^−1^	Translation rate
*c*_*R*_	µM ribosomes	Concentration of ribosomes
*h*	nm	Grid spacing
*p*	s^−1^	Probability density
*t*	s	Time
*x*_*i*_	–	Coordinate
*v*	m s^−1^	Velocity
*D*	M^2^ s^−1^	Diffusion coefficient
*N*	(µm^3^)^−1^	Molecule concentration
*d*	s^−1^	Jump probability density
*α*	s^−1^ µm^−3^	Volumetric jump probability density
*r*	–	Random number
*k*_*diss*_	s^−1^	Dissociation constant

### Diffusion model

#### Grid

For the sake of simplicity, the reaction space was discretized into a 3-D lattice with equidistant spacing between grid points.

#### Derivation of jump probability

The probability that a molecule will jump between two grid points can be derived from the Smoluchowski equation (Eq. 1) with n $p\left( {\vec{x},t} \right)$ as the probability density function of a random variable *X*.

1 $$\frac{{\partial p\left( {\vec{X},t} \right)}}{\partial t} = - \nu \frac{{\partial p\left( {\vec{X},t} \right)}}{{\partial x_{i} }} + D\frac{{\partial^{2} p\left( {\vec{X},t} \right)}}{{\partial x_{i}^{2} }}$$

Neglecting convection (*v* = 0) and regarding a single dimension results in the following equation:

2 $$\frac{{\partial p\left( {\vec{X},t} \right)}}{\partial t} = D\frac{{\partial^{2} p\left( {\vec{X},t} \right)}}{{\partial x^{2} }}$$

In addition, discretizing the partial differential equation with central differences results in an ordinary differential equation,

3 $$\frac{{dp\left( {\vec{X},t} \right)}}{dt} = D\frac{{p_{i + 1} - 2p_{i} + p_{i - 1} }}{{\Delta x^{2} }},$$

where *N*_*i*_ describes the average number of molecules currently resting on position *j*. The mass balance of *N*_*i*_ is therefore:

4 $$\frac{{dN_{i} }}{dt} = \frac{D}{{\Delta x^{2} }} \left( {N_{i + 1} - 2 N_{i} + N_{i - 1} } \right).$$

**Fig. 6:**
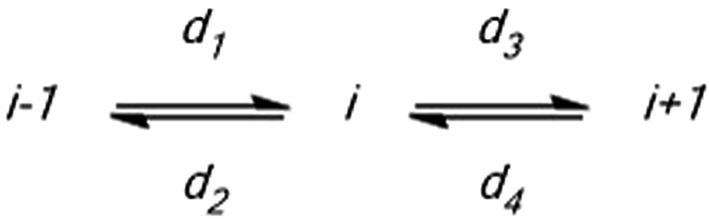
Diffusive transport reaction constants *d*_*i*_ for diffusive motion between point *i* and two adjacent points *i *± *1*

Based on the reaction scheme in Fig. 6, with the jump probabilities (*d*_*i*_) between the adjacent grid points *i *− *1*, *i,* and *i *+ *1*, a net reaction can be derived as shown in Eq. 5,

5 $$\begin{aligned} \frac{{dN_{i} }}{dt} = d_{1} + d_{4} - d_{2} - d_{3} \hfill \\ {\text{With}}\;d_{i} = d N_{i} \hfill \\ \end{aligned}$$

6 $$\begin{aligned} \frac{{dN_{i} }}{dt} = d \left( {N_{i - 1} - 2 N_{i} + N_{i + 1} } \right) \hfill \\ \widehat{ = }\frac{D}{{\Delta x^{2} }}\left( {N_{i - 1} - 2 N_{i} + N_{i + 1} } \right) \hfill \\ \end{aligned}$$

The specific jump probability can therefore be calculated based on the diffusion coefficient and the distance between the grid points as follows:

7 $$d = \frac{D}{{\Delta x^{2} }}$$

Equation 8 calculates the probability that molecules will jump between two grid points, and it can be used to calculate the jump probabilities for all three dimensions.

#### Random walk

The foundation of this model is a lattice-based random walk. The molecules that are considered are ternary complexes made from tRNA, EFTu, and ribosomes. The transport of ribosomes is neglected due to their much lower diffusion coefficient, and the reaction space is a 3-D lattice with a grid distance of *h*. A collision between two molecules only occurs if they touch each other, which implies that their distance is closer than the sum of their radii. A collision on the grid is defined as two molecules being on the same point. Thus, $N_{a}$ was chosen to be equal to the sum of the radii of ternary complexes and ribosomes. The probability (*α*_*i*_) of moving from a point towards one of its six adjacent points is defined as

8 $$\alpha_{travel,i} = \frac{D}{{h^{2} }} N_{i}$$

where *D*_*i*_ is the diffusion coefficient of molecule *i* and *N*_*i*_ is the number of molecules (*i*) at this position. The direction in which the molecule travels is chosen randomly with probabilities set equally (1/6 for each direction) using Gillespie’s direct stochastic simulation algorithm [8]. Total diffusion probability *α*_0_ is defined as the sum of all *α*_*i*_

9 $$\alpha_{0} = \mathop \sum \limits_{i = 1}^{{N_{molecules} }} \mathop \sum \nolimits \alpha_{travel,i}$$

and $N_{molecules}$ is the number of different possible ternary complexes. The time increment is calculated as shown in Eq. 10:

10 $$\tau = \frac{1}{{\alpha_{0} }}\ln \left( {\frac{1}{{r_{1} }}} \right)$$

In addition, the traveling molecule species *j* is selected as the smallest integer that fulfills Eq. 11,

11 $$\mathop \sum \limits_{i = 1}^{j} \alpha_{i} > r_{2} \alpha_{0}$$

where *r*_1_ and *r*_2_ are random numbers from a uniform distribution between zero and unity, calculated with a Mersenne Twister [13, 19]. After each step, the global time (*t*) is incremented by *τ*. The next necessary index is the molecule index *k*, which describes the discrete molecule of species *j* that wanders; it is chosen as a random integer between 1 and *N*_*j*_ (*N*_*j*_ is the number of molecules of species *j*).

The chosen molecule *N*_*j*_ (*k*) then travels to one of its neighbor grid points, and the direction follows a random distribution between 1 and 6. Each movement is followed by a check-up where the new position *N*_*new*_ is scanned for possible reaction partners (in this case a ribosome with a matching anticodon). If there is no reaction partner, the algorithm moves to the next increment. If there is a possible reaction partner, the molecule update sequence is identified where the ternary complex is split and the free EFTu is instantly bound to one of the free tRNAs, forming a new ternary complex that is randomly relocated in the reaction space. The ribosome elongates one codon and cannot react for a timespan of *t*_*cat*_, which correlates to the time required to refold the ribosome and prolong the peptide sequence. It is calculated as the reciprocal of the maximum specific elongation rate (24 amino acids per ribosome per second according to Arnold et al. [1]). During this idle time, the ribosome is not allowed to react further.

An additional phenomenon included in this model is the dissociation of ternary complexes. The probability of dissociation follows first-order kinetics as described in Eq. 12

12 $$\alpha_{diss,i} = N_{i} k_{diss}$$

where *k*_*diss*_ is the reaction constant for the dissociation of ternary complexes and is set to 1 s^−1^, [7]. This expansion leads to a new equation for the total probability:

13 $$\alpha_{0} = \mathop \sum \limits_{i = 1}^{{N_{molecules} }} \mathop \sum \nolimits \alpha_{travel,i} + \mathop \sum \limits_{i = 1}^{{N_{molecules} }} \mathop \sum \nolimits \alpha_{diss,i}$$

If the index *j* in Eq. 11 is selected as higher than the probability of travel, dissociation occurs instead of travelling. Dissociation is followed by choosing the species *j* and molecule *N*_*j*_ (*k*). This molecule is then split and its underlying tRNA is added to the pool of free tRNA. The released EFTu binds a randomly selected free tRNA and relocates to a random grid point.

The initial molecule distribution is set randomly for ternary complexes and ribosomes and the initial states of the ribosomes are uniformly distributed throughout the entire sequence. Translation, termination, and initiation were omitted in this model and ribosomes reaching the end of the sequence were set to the first codon. Furthermore, the entire calculation is nested in a loop with the stop criterion set to 5000 successful elongation steps. The specific elongation rate is calculated as the slope of step number over their respective time points. The resulting slope (elongations per second or amino acids per second) is normalized on the ribosome count, resulting in the specific elongation rate (elongations/amino acids per second per ribosome). The corresponding error is based on the deviation between ten different simulation runs with varying seeds for the random number generator.

#### Initial conditions

The numbers of actively translating ribosomes (ribosomes during elongation) and EFTu are shown in Table 2 and the number of tRNAs for the different species are shown in Table 3. For simulation purposes, a reaction volume of 0.064 µm^3^ was chosen, which results in at least 80 tRNAs in the reaction volume and an even number of grid points. The diffusion coefficient for all ternary complexes was set to $D = 2.567\,10^{ - 12} {\text{m}}^{2} {\text{s}}^{ - 1}$ [6] and diffusive transport for ribosome-mRNA complexes was excluded due to the drastically lower diffusion coefficient compared to ternary complexes.

**Table 2:** Initial number of actively translating ribosomes and EFTu during the simulation of translation in a reaction compartment with $V = 0.064\,\upmu{\text{m}}^{3}$

Molecule	Number	Source
Ribosomes	1044	Rudorf and Lipowsky [18]
EFTu	9122	Rudorf and Lipowsky [18]

The number of molecules was calculated for concentrations at a given growth rate of $\mu = 1.1\,{\text{h}}^{ - 1}$

**Table 3:** Quantity of tRNAs during the simulation of translation in a reaction compartment with $V = 0.064\,\upmu{\text{m}}^{3}$

tRNA species	Quantity	tRNA species	Quantity	tRNA species	Quantity
Ala1B	675.2	Gly3	764.6	Pro3	98.3
Ala2	122.9	His	129.1	Ser1	269.0
Arg2	916.1	Ile	729.2	Ser2	52.8
Arg3	87.1	Leu1	821.7	Ser3	208.1
Arg4	125.6	Leu2	181.9	Ser5	141.8
Arg5	94.8	Leu3	122.9	Thr1	21.6
Asn	235.1	Leu4	372.3	Thr2	102.9
Asp1	464.0	Leu5	140.7	Thr3	187.3
Cys	271.3	Lys	336.5	Thr4	192.3
Gln1	122.2	Met	158.0	Trp	159.9
Gln2	195.4	Phe	180.8	Tyr12	378.9
Glu2	929.6	Pro1	106.0	Val1	731.9

Derived from Dong et al. [5] for growth rate of 1.1 per hour

### Costs to synthesize nucleotides

The costs of the nucleotides that are necessary for RNA synthesis are described in Table 4. Amino acid costs were taken from Kaleta et al. [11] where precursor costs were excluded. Analysis of the sequence and the costs of each amino acid of EFTu led to production costs of 1989 ATP equivalents to synthesize the amino acids and 1576 ATP equivalents for translation (4 ATP equivalents per step). The overall synthesis cost for EFTu was therefore 3565 ATP equivalents per molecule. Ribosomes are composed of three types of RNA (5S, 16S, and 23S), which have lengths of 120, 1542, and 2906 nucleotides, respectively. These species and their respective sequences result in costs of 42,934 ATP equivalents to synthesize the rRNA of the ribosome. The protein content of ribosomes consists of 7459 AA with an average amino acid distribution from Spahr [22], which leads to costs of 35,689 ATP equivalents to synthesize the amino acids and 29,836 ATP equivalents for translation. Thus, ribosome synthesis requires 108,461 ATP equivalents per single molecule. tRNAs are an average of 76 nt in length, and an average sequence costs approximately 700 ATP equivalents per tRNA.

**Table 4:** Energy costs to produce the five different nucleotides based on the stoichiometric pathways of *E. coli*

	ATP equivalents
UTP/TTP	6
CTP	7
ATP	11
GTP	12
